# Does Reading-While-Listening Facilitate Reading in Older Adults? Evidence from Eye Movements

**DOI:** 10.3390/bs16061020

**Published:** 2026-06-18

**Authors:** Lin Li, Chenxi Wang, Yaning Ji, Jiaxin Du, Kevin B. Paterson

**Affiliations:** 1 Key Research Base of Humanities and Social Sciences of the Ministry of Education, Academy of Psychology and Behavior, Tianjin Normal University, Tianjin 300387, China; wangcx@stu.tjnu.edu.cn (C.W.); jiyaning@stu.tjnu.edu.cn (Y.J.); djx@stu.tjnu.edu.cn (J.D.); 2 Faculty of Psychology, Tianjin Normal University, Tianjin 300387, China; 3 School of Psychology and Vision Sciences, University of Leicester, Leicester LE1 7RH, UK

**Keywords:** aging, reading-while-listening, eye movements, Chinese reading, word segmentation

## Abstract

Previous research suggests receiving a concurrent auditory version of text during reading, i.e., reading-while-listening, improves eye-movement behavior in less skilled readers, while disrupting skilled reading. We extended this approach to older adults (65+ years) to see whether they benefit similarly. The experiment was conducted in Chinese, comparing young and older adults from an earlier reading-only study with new, closely matched groups presented with the same stimuli in a reading-while-listening task. Stimuli comprised either sentences containing a temporary ambiguity or matched unambiguous control, with plausibility manipulated for control target words and the incorrect interpretation of the ambiguity. At the sentence level, older adults showed differences across reading modes, with faster reading during reading-while-listening, whereas younger adults showed no evidence that reading-while-listening benefited reading, with some evidence it was disruptive. At the word level, both groups produced control-word plausibility effects, but neither showed plausibility effects for ambiguous targets, with no influence of reading mode. The findings suggest differential effects of reading-while-listening, potentially facilitating reading in older adults while yielding no benefit and some disruption in younger adults. As neither group had difficulty processing the ambiguity, no conclusions can be drawn about local ambiguity resolution. Nevertheless, the results suggest possible benefits of reading-while-listening for older adults.

## 1. Introduction

Reading is a fundamental cognitive skill that supports independence, communication, and access to information across the lifespan. However, substantial evidence indicates that reading performance declines with healthy aging. Compared with young adults (aged 18–30 years), older readers (aged 60+ years) typically exhibit slower reading, longer fixation durations, more regressions, and reduced efficiency in eye-movement control (Kliegl et al., 2004; S. Li et al., 2018; L. Li et al., 2024; Liu et al., 2020; Pagán et al., 2025; Rayner et al., 2006; Wang et al., 2018a, 2018b; Warrington et al., 2018; Zhao et al., 2019, 2021; for a meta-analysis, see J. Zhang et al., 2022). These age-related changes have been linked to declines in visual processing (Owsley, 2011; see also He et al., 2021; F. Xie et al., 2019), attentional control, and working memory (Kliegl et al., 2004; Gordon et al., 2016). At the same time, older adults often make use of compensatory strategies to maintain successful comprehension (Stine-Morrow et al., 2010; see also Payne & Stine-Morrow, 2014).

Despite these challenges, comprehension in older adults is often relatively well preserved (Riffo et al., 2024; Gordon et al., 2016; Choi et al., 2017; Steen-Baker et al., 2017), suggesting that older readers adapt their processing strategies to offset declines in lower-level perception and cognition (Rayner et al., 2006; Stine-Morrow, 2007; Stine-Morrow et al., 2008, 2010; Payne et al., 2012). An important question, therefore, is how reading can be supported in aging populations to improve reading efficiency without compromising comprehension. Identifying effective forms of support may be particularly important in Chinese, where characteristics of the writing system place substantial demands on visual and lexical processing (X. Li et al., 2015; Zang et al., 2011; L. Li et al., 2024). Chinese uses visually complex characters and presents text without spaces between words (for reviews, see X. Li et al., 2015; L. Li et al., 2024; Zang et al., 2011). Although some words consist of a single character, many comprise two or more characters, with multicharacter compounds accounting for more than 70% of words (Beijing Language Institute, 1986). Consequently, readers must determine word boundaries online during reading (Liversedge et al., 2016; X. Li & Pollatsek, 2020; Zhou & Li, 2021), a process that may be especially challenging for older adults.

One promising avenue for supporting reading in older adults may be to provide additional information across modalities, for example, by pairing visual text with concurrent auditory input. Much of the research on multisensory language processing has focused on the contribution of visual speech cues, particularly facial and lip movements, to spoken-language comprehension (for reviews, see Peelle & Sommers, 2015; Venezia et al., 2015). Across this literature, performance on tasks such as phoneme identification, word recognition, and sentence comprehension is typically enhanced in audiovisual conditions relative to unimodal presentation (see, e.g., Chauvin et al., 2024; Smayda et al., 2016; Sommers et al., 2005; Stevenson et al., 2015). These findings demonstrate that integrating auditory and visual information can facilitate access to linguistic meaning, raising the possibility that concurrent auditory input may also support sentence comprehension.

Other research, primarily in second-language (L2) reading, has examined whether reading comprehension benefits from the concurrent auditory presentation of the text being read (Brown et al., 2008; Chang & Millett, 2015; Webb & Chang, 2012, 2014; Y. Xie et al., 2025; see also Gerbier et al., 2018). Findings from this reading-while-listening literature show that L2 readers perform better on measures of text comprehension, recall, and vocabulary learning when text is presented both visually and auditorily than when it is read without accompanying auditory input. Similar benefits have been reported in multimedia-learning contexts, where listening to a spoken narration while reading on-screen text can enhance comprehension and retention relative to reading alone (Fenesi et al., 2015). Particularly relevant to the present study is the eye-movement investigation by Conklin et al. (2020), which compared L1 and L2 readers under reading-only and reading-while-listening conditions. In the reading-only condition, L2 readers showed slower and more effortful processing than L1 readers, reflected in more and longer fixations and reduced word skipping. Under reading-while-listening, however, the eye-movement behavior of the two groups became more similar. This appeared to reflect a slowing effect of concurrent auditory input on L1 reading, with relatively little additional effect on the already slower reading patterns of L2 readers. These findings demonstrate that eye movements are sensitive to the processing consequences of reading-while-listening and suggest that the effects of auditory support may depend on readers’ existing level of reading proficiency. However, it remains unclear whether similar effects extend to native-language reading in older adults, particularly during online processing.

Accordingly, the present experiment employed a reading-while-listening paradigm combined with eye-movement measures to determine whether supplementary auditory input supports Chinese reading in older adults. Specifically, we examined whether presenting Chinese sentences auditorily while participants read them facilitated older adults’ reading behavior relative to a reading-only condition. Eye movements were used to provide a detailed account of how concurrent auditory input affected reading performance in both young and older adults. These measures offer insight into both global sentence-level reading behavior and the moment-to-moment processing of individual words (Rayner et al., 1998; Rayner, 2009), making them well suited to assessing both overall reading performance and local word-processing effects, including the processing of segmental ambiguities in Chinese.

A central question for the present research was whether reading-while-listening would produce changes in older adults’ eye-movement behavior similar to Conklin et al.’s (2020) findings for L2 readers. Older adults often show slower and more effortful eye-movement patterns than young adults, and in Chinese, this may partly reflect the additional demands of word segmentation in naturally unspaced text. If supplementary auditory input supports lexical access to individual words, older readers may show more efficient eye-movement behavior during reading-while-listening than during reading only.

Conklin et al. (2020) also found evidence that reading-while-listening increased processing difficulty for L1 readers, suggesting that supplementary auditory input is not necessarily beneficial for all readers. Rather, its effects may depend on readers’ existing level of reading skill. For less efficient readers, auditory input may support lexical access and sentence comprehension, whereas for skilled readers, it may be unnecessary and may interfere with an already efficient reading process. This possibility is relevant to the young adult readers in the present study, who, like Conklin et al.’s L1 readers, may already process sentences efficiently in the reading-only condition. We therefore considered the possibility that reading-while-listening would benefit older readers, but provide little benefit, or even produce interference, for skilled young adult readers.

In addition to its effects on overall reading performance, reading-while-listening may influence local word-segmentation processes. To examine this possibility, we investigated the processing of temporary segmental ambiguities, which arise when the same sequence of Chinese characters permits multiple possible segmentations. Resolving such ambiguities requires the use of contextual information (Q. Zhang & Li, 2026; Huang et al., 2021) and may be particularly demanding for older adults (L. Li et al., 2024; Yi et al., 2023). This raises the question of whether concurrent auditory input can support word segmentation during reading, and whether any such support differs across age groups.

The sentence stimuli included ambiguous sentences and matched unambiguous control sentences. In the ambiguous condition, sentences contained a three-character word whose first two characters could also form a standalone word (e.g., “地形图”, “topographic map”, where “地形” means “terrain”). This created a temporary ambiguity between an embedded-word interpretation and a whole-word interpretation. In the control condition, the same three-character word was replaced by the corresponding unambiguous two-character word, allowing a direct comparison between processing of the embedded word when it appeared independently and when it formed part of a larger lexical unit. Following previous work (Zhou & Li, 2021; L. Li et al., 2024), we manipulated contextual plausibility by varying the immediately preceding verb. Thus, the two-character control target and the embedded word within the ambiguous sequence were either plausible (观察地形, “survey terrain”) or implausible (打开地形, “open terrain”), while the three-character word remained plausible in both contexts (观察/打开地形图, “survey/open topographic map”). Plausibility effects for the control targets provided a manipulation check, whereas plausibility effects for the embedded word in the ambiguous condition would indicate that readers initially identified and processed the embedded-word representation. The manipulation, therefore, allowed us to assess whether embedded-word activation varied as a function of age and the presence of concurrent auditory input.

Based on Conklin et al. (2020), we expected reading-while-listening to affect younger and older adults differently. If concurrent auditory input provides additional support for reading, older adults should show more efficient eye-movement behavior during reading-while-listening than during reading only, reflected in shorter reading times and reduced evidence of effortful processing. In contrast, young adults were expected to show little benefit and potentially some cost, resulting in slower or more effortful sentence reading due to interference from the redundant auditory input.

At the target-word level, we expected young adults to segment the ambiguous sequence correctly as a whole word, without initially accessing the embedded two-character word. They should therefore show plausibility effects for the unambiguous control sentences, but no corresponding embedded-word plausibility effects in the ambiguous sentences. Predictions for older adults were less clear. If older readers experience greater difficulty with word segmentation, they may show evidence of accessing the embedded word, producing an embedded-word plausibility effect in ambiguous sentences. However, older readers did not produce such effects in the reading-only condition, suggesting that they segmented these ambiguities similarly to young adults. The key question was therefore whether a similar pattern would be observed during reading-while-listening, and whether concurrent auditory input would influence the activation of embedded-word representations during ambiguity resolution.

## 2. Materials and Methods

### 2.1. Transparency and Openness

Ethics statement. The research was conducted in accordance with the principles of the Declaration of Helsinki (World Medical Association, 2013) and approved by the ethics committee in the Academy of Psychology and Behavior at Tianjin Normal University, Protocol Number APB2025040302, study entitled: “Word Segmentation in older adults: The role of reading-while-listening modality”.

Sentence stimuli, datasets and analytic code for analyses conducted in R are publicly available via: https://osf.io/94zpu (accessed on 1 May 2026).

**Figure 1:**
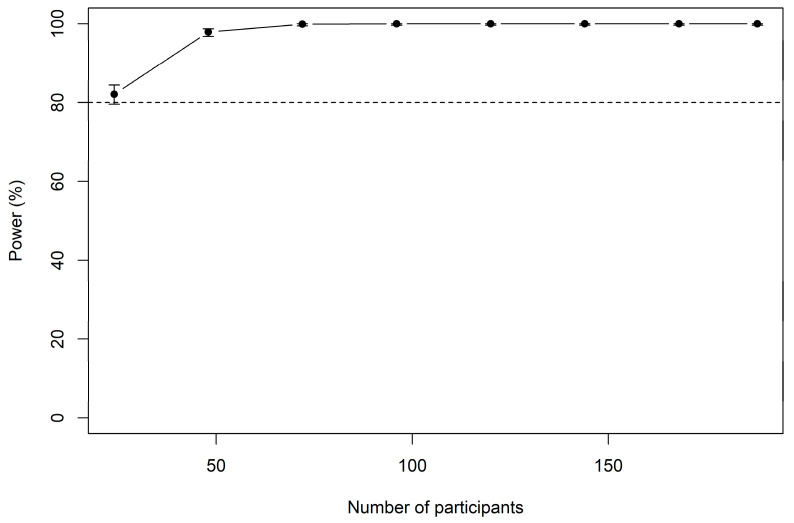
Power curve for the Age × Reading Condition interaction effect.

Participants. Simulation-based power analyses indicated that the observed Age × Reading Condition interaction could be detected with adequate statistical power (see Figure 1). Each data point represents power estimated from 1000 simulations. Error bars indicate 95% confidence intervals. The dashed horizontal line indicates the 80% power threshold. The actual sample size used in the present study (N = 183) substantially exceeded the sample sizes associated with conventional power levels. In addition, a sensitivity analysis based on the present sample size is reported in the Supplementary Materials, indicating that the study was sufficiently powered to detect small-to-moderate interaction effects.

As our main goal was to examine differences between young and older adults, we recruited a larger sample than that suggested by the power analysis. Accordingly, the experiment combined existing young and older adult data from L. Li et al. (2024) for the reading-only condition with newly collected young and older adult data for the reading-while-listening condition. The existing sample consisted of 48 young adult students aged 19–25 years (*M* = 21.7 years, 30 females) and 36 older adults aged 65–77 years (*M* = 69.4 years, 21 females), who read the sentence stimuli in the reading-only condition while their eye movements were recorded.

The newly collected data were from 60 undergraduate students aged 18–25 years (*M* = 20.9 years, 52 females) and 39 older adults aged 60–81 years (*M* = 68.9 years, 25 females). All young adults were recruited from Tianjin Normal University in Tianjin, China, whereas all older adults were recruited from the local Tianjin community. Participants were paid a small sum (50 RMB) for their participation. All had normal or corrected-to-normal vision, reported normal hearing, and were native Chinese readers.

All older adults were assessed for cognitive functioning using the Montreal Cognitive Assessment (Beijing version; standard exclusion criterion: score < 26/30; Nasreddine et al., 2005), visual acuity using a tumbling E eye chart (better than 20/40 Snellen; H. R. Taylor, 1978), and vocabulary knowledge and working memory using the Vocabulary Knowledge and Digit Span subtests of the Chinese version of the Wechsler Adult Intelligence Scale, third edition (WAIS-III; Wechsler, 1997; Wechsler et al., 2002). The characteristics of the older adult sample are presented in Table 1.

### 2.2. Stimuli and Design

The sentence stimuli comprised 80 sets of sentences constructed by L. Li et al. (2024). Following Zhou and Li (2021), L. Li et al. used three-character words (e.g., “地形图”, meaning “topographic map”) in the ambiguity condition and corresponding two-character target words (e.g., “地形”, meaning “terrain”) in control sentences with the same sentence structures (see Table 2 for examples). The target words in the ambiguity condition were temporarily ambiguous because their initial two characters formed a standalone word identical to the two-character control target. We refer to this initial two-character string within the three-character target word as the embedded word.

Each sentence also contained one of two interchangeable verbs immediately preceding the target word. In the control condition, the two-character target word was combined with the preceding verb either plausibly (e.g., “观察地形”, “survey terrain”) or implausibly (e.g., “打开地形”, “open terrain”). In the ambiguity condition, the embedded words were identical to the control target words and so were either plausible or implausible in the verb context. However, the 3-character word as a whole was selected to be plausible in both verb contexts. This manipulation, therefore, created a temporary ambiguity in the experimental sentences, such that if readers processed the first two characters of the target word as a standalone word, this would produce an equivalent plausibility effect as the control stimuli (see Table 3 for characteristics of the Pre-target Verbs).

Prior to the experiment, we normed the sentence stimuli for plausibility. There were no differences in plausibility ratings among sentences containing a three-character target word with a plausible embedded word, a three-character target word with an implausible embedded word, or a plausible two-character target word (young adults: *ts* < 1.18, *ps* > 0.24; older adults: *ts* < 0.74, *ps* > 0.46). This indicates that, in the offline rating task, plausibility judgments were not affected by the plausibility of the verb-embedded word combination. As expected, however, ratings were lower for sentences containing an implausible two-character target word than for those in the other conditions (young adults: *ts* > 22.38, *ps* < 0.001; older adults: *ts* > 17.41, *ps* < 0.001). This shows that participants were sensitive to the plausibility of the verb-target word combination in the control condition, confirming the effectiveness of the manipulation. This pattern was comparable across age groups, indicating that young and older adults made similar plausibility judgments.

We conducted a cloze task (W. L. Taylor, 1953) to assess target word predictability and ensure that any observed effects could be attributed to differences in plausibility rather than predictability. Ten young adults and 10 older adults were asked to provide written continuations for sentences truncated immediately before the target word. Cloze probabilities for the target words were close to zero, indicating that they were equally unpredictable from the preceding context, with no evidence of age-group differences (*ts* < 0.83, *ps* > 0.41). This suggests that any eye-movement effects observed in the experiment can be attributed to plausibility rather than predictability in both young and older adult groups. None of the participants in the norming studies took part in the eye-movement experiment.

The sentences were split into four different lists. Each list contained four practice sentences, 80 experimental sentences, and 20 filler sentences (all plausible to minimize participants’ awareness of the experimental purpose). The 80 experimental sentences comprised four different conditions with 20 sentences per condition. Using a Latin square, sentences were evenly distributed across lists to ensure each list contained one version per condition while keeping equal condition representation per list. Experimental and filler sentences were displayed in randomized order, with 25% of the sentences followed by content-related comprehension questions, requiring a yes or no response.

In the reading-only condition, no auditory stimuli were presented. In the reading-while-listening condition, participants listened to auditory stimuli generated using an AI-based text-to-speech platform (iFLYTEK, https://xunfeizhizuo.ai-tab.cn, access date 15 March 2025). Sentences were converted into spoken Mandarin using a male voice designed to approximate a natural speaking style. The speech rate was controlled at 280–300 words per minute (Meng, 2006), and each sentence was delivered with a neutral intonation. All audio files were standardized in WAV format with a sampling rate of 44,100 Hz, stereo (two-channel) output, 32-bit resolution, and a bit rate of 384 kbps, ensuring a consistent experience across trials. Across the experimental sentence recordings, the mean audio duration was 4630 ms (SD = 567).

The experiment employed a mixed design with four factors: a between-participants factor of age group (young adults, older adults), a between-participants factor of reading condition (reading-only, reading-while-listening), a within-participants factor of target word type (ambiguous word, control target), and a within-participants factor of target word plausibility (plausible, implausible), with plausibility for the ambiguous word defined in terms of its embedded word plausibility.

The young adults in each reading condition were drawn from the same undergraduate student population. The older adults in the two reading conditions were also drawn from the same local population. However, as the older adult sample was expected to be more heterogeneous, participants assigned to the two reading conditions were carefully matched on age, educational background, visual acuity, and cognitive abilities. Accordingly, there were no significant differences between the two older adult groups in age (*t* = 0.53, *p* > 0.05, 95% CI [−1.51, 2.60]), years of education (*t* = 0.61, *p* > 0.05, 95% CI [−0.72, 1.35]), visual acuity (*t* = −0.86, *p* > 0.05, 95% CI [−0.04, 0.02]), Digit Span scores (*t* = −1.38, *p* > 0.05, 95% CI [−1.18, 0.21]), vocabulary scores (*t* = −1.10, *p* > 0.05, 95% CI [−6.16, 1.79]), or MoCA scores (*t* = 1.27, *p* > 0.05, 95% CI [−0.21, 0.94]).

Young and older adults within each reading condition were pseudo-randomly assigned to sentence stimulus lists such that equal numbers of young and older participants were allocated to each list across reading-only and reading-while-listening conditions.

### 2.3. Apparatus and Procedure

Eye movements during reading were recorded using an EyeLink 1000 Plus eye tracker (SR Research, Ottawa, ON, Canada). Participants read binocularly, although only gaze position from the right eye was recorded. The eye tracker provided high spatial resolution (<0.01° root mean square) and a sampling rate of 1000 Hz. Head position was stabilized using a forehead and chin rest. Stimuli were presented on a 24-inch monitor (1920 × 1080 pixels, 150 Hz refresh rate). Text materials were displayed in Song font as black characters (RGB: 0, 0, 0) on a gray background (RGB: 220, 220, 220), with each character subtending 53 × 53 pixels. At a viewing distance of 75 cm, each character occupied approximately 1° of visual angle. Auditory stimuli were presented through the computer’s built-in speakers at a comfortable sound level of approximately 70 dB (Inhoff et al., 2004), with no background noise.

All participants provided written informed consent before the experiment began. Each participant was tested individually and received instructions before starting the task. At the beginning of the experiment, a three-point horizontal calibration procedure was carried out along the same line on which each sentence was presented, ensuring spatial accuracy of 0.35° or better for all participants. Before each trial, calibration accuracy was checked, and recalibration was performed when necessary to maintain precision throughout the experiment.

**Figure 2:**
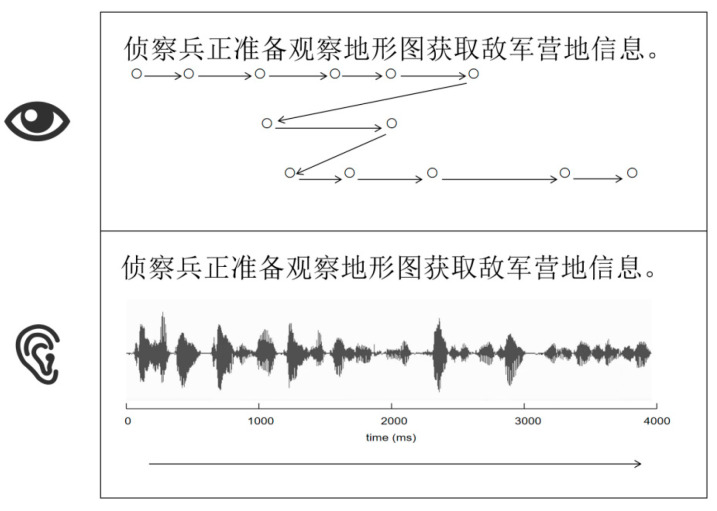
In the reading-while-listening condition, participants look and listen at the same time. The translation of the sentence is “The scout is preparing to survey a topographic map to get information about the enemy camp.”.

In the reading-only condition, participants read each sentence without accompanying auditory input. In the reading-while-listening condition, participants read each sentence while simultaneously hearing it spoken aloud. The auditory presentation was not synchronized with participants’ eye movements or reading speed. At the start of each trial, a fixation square equal in size to one character appeared on the left side of the screen. Once the participant fixated on this location, the sentence was presented, with the first character replacing the square. In the reading-while-listening condition, sentence onset also triggered the onset of the audio presentation (see Figure 2). Participants were instructed to read each sentence for comprehension. They were also told to press the spacebar to proceed to the next trial as soon as they finished reading the sentence, regardless of whether the audio had finished playing. On 25% of trials in both conditions, a visually presented comprehension question followed the sentence and required a “yes” (f key) or “no” (j key) response. The full procedure lasted approximately 40 min per participant.

Importantly, as part of our effort to match the reading-only and reading-while-listening groups as closely as possible, we collected data from the two reading modes in the same laboratory, using the same eye-tracking equipment, experimental protocols, data-analysis procedures, and stimuli. The studies were also conducted by the same two experimenters.

## 3. Results

### 3.1. Data Analysis Procedure

Eye-movement data were first screened using a standard procedure that excluded fixations on target words shorter than 80 ms or longer than 1200 ms. Following this procedure, 3.1% of trials were removed for young adults and 6.8% for older adults. The remaining data were analyzed in RStudio (version 4.3.2, R Core Team, 2024). Linear mixed-effects models (LMEMs) were fitted to fixation-duration measures using the lme4 package (Bates et al., 2011), and generalized linear mixed-effects models were used for binary outcome measures (word-skipping rate, regression rate, and comprehension question accuracy). *p* values were computed using the lmerTest package (Kuznetsova et al., 2014).

Following Zhou and Li (2021) and L. Li et al. (2024), we used LMEMs to conduct theoretically motivated contrast analyses (Schad et al., 2020) separately for young and older adults. Specifically, we tested two contrasts: (a) the effect of control-word plausibility, and (b) the effect of embedded-word plausibility. Control-word plausibility and embedded-word plausibility were entered as fixed effects in separate models for young and older adults, with random intercepts and slopes included where appropriate (Baayen et al., 2008). Continuous variables (fixation durations) were log-transformed (Wagenmakers et al., 2012). Contrasts were coded using the contr.sdif function from the MASS package (Venables & Ripley, 2002).

Following Barr et al. (2013), the initial analyses included a maximal random-effects structure, with participants and items specified as crossed random effects. If the full model failed to converge, the random-effects structure was simplified systematically, beginning with the item random effects: random-effect correlations were removed first, followed by random slopes, before applying the same procedure to the participant random effects (Matuschek et al., 2017). This process continued until model convergence was achieved. No singular fits were detected in any of the final converged models.

We used eye-movement measures that are informative for both sentence-level and word-level analyses (Rayner et al., 1998, Rayner, 2009). Sentence-level analyses were used to examine between-group differences in the effects of reading condition on global eye-movement behaviors. For these analyses, we examined effects in overall sentence reading time (SRT), average fixation duration (AFD), number of fixations (NF), number of regressions (NR; backward eye movements in the text), and the average length of forward-directed saccades (AFS; i.e., mean length of forward-directed eye movements, calculated as number of characters per saccade).

Word-level analyses were used to assess between-group differences in the effects of reading condition on ambiguity processing at the target word region. This region comprised either the two-character control target word or the three-character ambiguous word. Following Zhou and Li (2021) and L. Li et al. (2024), we analyzed effects at the critical region using measures sensitive to both early, first-pass processing of the ambiguity, and its later processing, Rayner (2009). For measures sensitive to early processing, we examined first-fixation duration (FFD), the duration of the first progressive fixation on a word, and gaze duration (GD), the sum of all first-pass fixations. For measures sensitive to later processing, we examined total reading time (TRT), the sum of all fixations on a word, and regressions-in (RI), the probability of making a regression back to the critical region.

### 3.2. Data Analysis

#### 3.2.1. Comprehension Accuracy

Young adults (reading-only: *M* = 94.7%; reading-while-listening: *M* = 98.2%, *p* < 0.001) and older adults (reading-only: *M* = 95.2%; reading-while-listening: *M* = 95.1%, *p* = 0.61) had comparably high accuracy rates > 94% in sentence comprehension. These indicated that both age groups could understand the sentences well in both reading-only and reading-while-listening conditions.

#### 3.2.2. Sentence-Level Analyses

Mean values and standard errors for sentence-level eye movements for young and older adults are presented in Table 4, and Table 5 summarizes the statistical results.

As noted earlier, the reading-only data were drawn from a previous study on age-related differences in reading. The results showed the expected age-group differences in global reading behaviors. Compared with young adults, older adults had longer sentence reading times, longer average fixation durations, made shorter forward saccades, and produced more regressions to earlier parts of the text. The central question for the present study was whether the reading-while-listening condition would influence these patterns of reading behaviors in the two age groups. The results showed significant interactions between age group and reading condition in sentence reading time, average fixation duration, number of fixations, number of regressions, and forward saccade amplitude. The older adults showed clear benefits for the reading-while-listening condition.

Relative to the reading-only condition, older adults in the reading-while-listening condition exhibited shorter sentence reading times, fewer fixations and regressions, and longer forward-directed eye movements, with no reliable difference in average fixation duration. By contrast, young adults in the reading-while-listening condition exhibited longer average fixation durations and shorter forward-directed eye movements relative to the reading-only condition, with no differences in sentence reading time, number of fixations, or number of regressions.

Sentence reading time provides the clearest overall index of the effect of reading condition, revealing shorter reading times for older adults in the reading-while-listening compared with the reading-only condition, but no corresponding effect for young adults. For older adults, this facilitation was accompanied by more efficient eye-movement behavior, as evidenced by longer forward eye movements and fewer fixations, as well as a reduced need to reread earlier text, as evidenced by fewer regressions. Importantly, these benefits occurred without any cost to comprehension accuracy.

By contrast, young adults exhibited changes in specific components of eye-movement behavior, including longer fixation durations and shorter forward eye movements in the reading-while-listening condition, consistent with increased processing difficulty. These changes did not translate into differences in overall sentence reading time. Notably, comprehension accuracy remained high across both conditions (reading-only: 94.7%; reading-while-listening: 98.2%), indicating that these changes in eye-movement behavior did not compromise overall understanding.

To further clarify the interaction between age group and reading condition, we examined age differences within each condition. In the reading-only condition, older adults showed substantially slower sentence reading times than young adults, along with longer fixation durations, more fixations and more regressions, indicating pronounced age-related differences in reading efficiency. By contrast, in the reading-while-listening condition, these age differences were attenuated. Although older adults remained slower overall, the magnitude of the age-related differences in sentence reading time and eye-movement measures was reduced. This pattern indicates that the interaction was primarily driven by improvements in older adults’ performance under reading-while-listening conditions, rather than any facilitative effects in young adults, thereby selectively reducing age-related disparities in reading performance.

#### 3.2.3. Word-Level Analyses

Means and standard errors for word-level eye movements are presented in Table 6, and the statistical findings are summarized in Table 7.

We employed multiple correlated eye movement measures as dependent variables when analyzing word-level effects. Von der Malsburg and Angele (2017) have highlighted risks in this approach, as using multiple dependent variables in eye-movement research can increase the risk of Type I errors. To mitigate this, they recommend specifying hypotheses in advance, interpreting effects from a single measure with caution unless predicted a priori, and applying Bonferroni or similar corrections (while noting that Bonferroni adjustments may be overly conservative for highly correlated fixation time variables).

For the word-level analyses, we focused on three main dependent measures (first-fixation duration, gaze duration, total reading time), as these are likely to capture effects occurring during either the initial, first-pass processing of the ambiguity, or later processing. Accordingly, we applied a Bonferroni correction to adjust for these three measures of interest, setting the threshold at *p* < 0.017. We report analyses separately for plausibility effects for the control words and the ambiguous words.

### 3.3. Control Word Plausibility

In the reading-only condition, young adults showed a control word plausibility effect in total reading time, with shorter reading times for plausible than implausible words (407 vs. 459 ms). No other measures showed a significant plausibility effect.

In the reading-only condition, the older adults also showed significant control word plausibility effect in total reading time (682 vs. 872 ms). In addition to this hypothesis-testing effect, the older adults also showed a plausibility effect in regressions-in probability, with a higher probability of making a regression back to the target word when it was implausible rather than plausible (43% vs. 35%). As RI was not subject to Bonferroni correction, this effect is reported as an exploratory observation.

In the reading-while-listening condition, the young adults showed a control word plausibility effect in both gaze duration (294 vs. 311 ms) and total reading time (442 vs. 515 ms). The older adults also showed a control word plausibility effect in total reading time (528 vs. 601 ms), and a similar regressions-in probability effect, with a higher regression probability for implausible than plausible words (32% vs. 26%). As indicated above, the RI effect is reported as an exploratory observation.

The effects indicate that both age groups exhibited sensitivity to control word plausibility, as confirmed by effects on total reading time surviving Bonferroni correction in all conditions. Additional effects on gaze duration and regressions-in are reported as exploratory observations. There was no indication that plausibility sensitivity differed across reading conditions.

### 3.4. Ambiguity Word Plausibility

With this analysis, we examined plausibility effects for the embedded word in the ambiguous three-character target word. In both the reading-only and reading-while-listening conditions, the young adults showed no effect of embedded word plausibility. Similarly, in both the reading-only and reading-while-listening conditions, the older adults showed no effect of embedded word plausibility after Bonferroni correction. Although a marginal effect was observed for older adults in gaze duration in the reading-while-listening condition (*p* = 0.045), this did not survive the corrected threshold and is not treated as confirmatory evidence. The findings therefore provide no evidence that either group accessed the embedded word analysis of the ambiguity in either reading-only or reading-while-listening conditions. This may be because readers do not access embedded word representations when processing this type of ambiguity. Alternatively, the present experiment may have been underpowered to detect such effects, the design may have been insufficiently sensitive, or the ambiguity may have been resolved very rapidly, such that the study was not sufficiently sensitive or powered to detect an effect.

## 4. Discussion

The present study investigated whether concurrent auditory input can support reading in older adults in a reading-while-listening task, and how such effects compare with those observed in young adults. Using eye-movement measures of both global sentence-level reading behavior and the local processing of segmental ambiguity, the results suggest that reading-while-listening differentially affects reading across age groups. At the sentence level, older adults showed more efficient reading behavior in the reading-while-listening condition relative to a matched group in the reading-only condition, whereas young adults showed no evidence of facilitation and some evidence of processing costs. At the word level, both age groups showed sensitivity to control-word plausibility across conditions, but neither showed evidence of processing difficulty associated with the segmental ambiguity. Consequently, the present data do not permit strong conclusions about whether reading-while-listening influences local ambiguity resolution. Nevertheless, the sentence-level findings suggest that concurrent auditory input can affect reading efficiency, particularly in older adults, while leaving its role in local ambiguity processing unresolved.

### 4.1. Older Adults Showed More Efficient Reading During Reading-While-Listening

The clearest finding was that older adults showed more efficient sentence-level eye-movement behavior in the reading-while-listening condition than in the matched reading-only condition. Specifically, older adults in the reading-while-listening condition had shorter sentence reading times, made fewer fixations and regressions, and produced longer forward-directed eye movements. This pattern suggests more efficient eye-movement control and a reduced need for rereading, consistent with more fluent reading. Older adults in the reading-only condition showed substantially slower and more effortful reading than young adults, replicating well-established age-related differences in reading performance (for a review, see J. Zhang et al., 2022). By comparison, this age-related disparity was smaller in the reading-while-listening condition. Although older adults remained slower overall, the reduced age difference suggests that concurrent auditory input may have selectively supported older adults’ reading performance. Crucially, these differences were observed without any cost to comprehension accuracy.

These findings are consistent with accounts of age-related declines in perceptual and cognitive efficiency (e.g., Owsley, 2011; Gordon et al., 2016), alongside compensatory mechanisms that support comprehension (e.g., Stine-Morrow et al., 2010; Payne & Stine-Morrow, 2014). The findings also suggest that the reading behavior of the older adults was superior in the reading-while-listening compared to the reading-only condition. This was consistent with older adults’ reading behavior benefiting from the concurrent auditory input in the reading-while-listening condition. However, the present data do not identify the specific mechanism through which concurrent auditory input may have supported older adults’ reading. One possibility is that the auditory stream helped pace readers’ progress through the text or provided attentional support during sentence processing. Consistent with this interpretation, supplementary analyses indicated that older adults completed reading before audio offset on only 37% of trials, compared with 72% for young adults, suggesting that older adults were more likely to be guided by the auditory stream throughout sentence processing (see Supplementary Materials, Section S1). Another possibility is that it provided additional lexical information, reducing uncertainty during word identification. The effects may also reflect strategic adaptation to the auditory input. The findings are broadly consistent with Conklin et al. (2020), who reported that L2 readers’ eye movements became more similar to those of skilled L1 readers in reading-while-listening conditions. Similarly, older adults’ eye movements in the present study became more similar to those of young adults. Thus, while the between-participants design limits causal inference, the findings suggest that reading-while-listening may support sentence-level reading behavior in readers who experience greater difficulty with written text. More broadly, the results align with evidence that older adults may derive particular benefit from multimodal input under conditions of increased processing demand (Chauvin et al., 2024; Smayda et al., 2016; Sommers et al., 2005), extending this possibility to sentence reading.

### 4.2. Reading-While-Listening Offers Little Benefit for Skilled Young Adult Reading

By comparison with the findings for the older adults, the young adults showed no evidence to suggest facilitative effects of supplementary auditory input in the reading-while-listening condition. Although the young adults’ overall sentence reading time was similar across reading-only and reading-while-listening conditions, those in the reading-while-listening condition made generally longer fixations and generally shorter forward-directed eye movements, consistent with more effortful reading. At the same time, their comprehension accuracy was not impaired, suggesting that these changes reflect processing costs rather than failures of understanding.

One possibility is that, for skilled readers, the supplementary auditory input is redundant and may impose additional processing demands. In particular, because the auditory input was not synchronized with eye movements, skilled readers may have experienced difficulty integrating the two sources of information. This may have disrupted their normal eye-movement behavior to some degree, potentially reflecting the additional demands of coordinating auditory and visual streams of linguistic information. Notably, this finding is broadly consistent with Conklin et al.’s (2020) observation that reading-while-listening disrupted the normal eye-movement behavior of skilled readers in a comparison of L1 and L2 readers. Taken together, the findings indicate that the effects of reading-while-listening are not uniform. Instead, they appear to depend on baseline reading efficiency, facilitating performance in readers who experience greater difficulty with written text (e.g., older adults and L2 readers) while introducing modest costs for more efficient readers (e.g., skilled young adults and L1 readers).

### 4.3. Word-Level Effects of Reading-While-Listening

At the word level, both young and older adults were sensitive to the plausibility of control words, indicating that both groups used contextual information online to evaluate target-word plausibility during reading. However, neither group showed effects of embedded-word plausibility for the temporarily ambiguous words, and this pattern was consistent across both reading conditions. Several explanations remain possible. The manipulation may have been insufficiently sensitive to detect embedded-word activation, the study may have been underpowered to detect subtle ambiguity effects, or readers may have resolved the ambiguity without strongly activating the embedded-word interpretation. Consequently, the present data do not provide a basis for assessing whether reading-while-listening influences local ambiguity resolution. More generally, the findings indicate that the effects of reading-while-listening observed in the present study were detectable at the level of global reading behavior, but leave its influence on local lexical processing unresolved. Future research should therefore examine its effects using manipulations known to influence lexical processing and eye-movement control, including word frequency, contextual predictability, and word length (see, e.g., Rayner, 2009).

### 4.4. Implications for Accounts of Aging and Reading

The present findings have several implications for theories of reading and cognitive aging. Most broadly, they suggest that the effects of reading-while-listening depend on characteristics of the reader rather than reflecting a uniform benefit of concurrent auditory input. Older adults showed more efficient sentence-level reading behavior under reading-while-listening conditions, whereas young adults showed little evidence of facilitation. This pattern is consistent with the view that supplementary input may be most beneficial when processing efficiency is constrained, whether by age-related changes in perceptual and cognitive functioning or by other factors that increase the demands of reading. More generally, the findings support accounts that emphasize compensatory mechanisms in aging (Stine-Morrow et al., 2010; see also Payne & Stine-Morrow, 2014), while suggesting that multimodal support may represent one route through which such compensation can occur. At the same time, the absence of comparable benefits in young adults highlights the importance of considering individual differences in reading proficiency and processing efficiency when evaluating the effects of reading-while-listening.

### 4.5. Limitations and Future Directions

Several limitations of the present study should be acknowledged. First, the auditory input was not synchronized with participants’ eye movements, which likely increased the demands of cross-modal integration. This may have contributed to the reduced processing efficiency observed in young adults, for whom the auditory input was potentially redundant. Future research should examine whether tighter temporal alignment, such as gaze-contingent or pace-controlled auditory presentation, enhances facilitation or reduces interference, particularly for skilled readers.

Second, this preliminary investigation employed a between-participants design, comparing carefully matched groups from reading-only and reading-while-listening conditions. Although this approach avoided repetition and carryover effects that would arise from presenting the same materials under both reading modes, it carries some risk due to potential heterogeneity in reading ability across groups. To minimize this risk, the groups were closely matched on key characteristics, and the experiments were conducted under near-identical conditions, using the same laboratory, equipment, protocols, stimuli, and experimenters. Nevertheless, because the data were collected separately, unmeasured cohort differences cannot be entirely excluded. Future research should therefore employ within-participants comparisons or alternative designs that permit stronger causal inferences about the effects of reading-while-listening.

A further limitation is that the present design does not allow us to determine the mechanism through which reading-while-listening influenced older adults’ reading behavior. The observed facilitation could reflect lexical support, attentional support, pacing effects, strategic adaptation, or a combination of these factors. Future studies should seek to distinguish among these possibilities more directly.

Another limitation concerns the absence of ambiguity effects. Because neither group showed evidence of difficulty processing the temporary ambiguity, the study does not provide a strong test of whether reading-while-listening influences local ambiguity resolution. The study may also have been underpowered to detect subtle ambiguity-related effects, limiting conclusions about local lexical processing. Future studies could therefore employ materials that more reliably induce ambiguity-related processing difficulty or examine effects on other determinants of lexical processing, such as word frequency, predictability, and length.

Finally, it remains unclear whether the present findings generalize beyond Chinese. Given the distinctive properties of Chinese orthography and text layout, especially the absence of explicit word boundaries, it will be important to determine whether similar effects occur in other writing systems. In addition, the higher proportion of female participants in the young adult reading-while-listening group limits the generalizability of the findings, and future research should consider more gender-balanced samples.

## 5. Conclusions

In summary, the present study suggests that reading-while-listening may enhance global reading efficiency in older adults without compromising comprehension, while offering little benefit and producing evidence of modest disruption in skilled young adult readers. These findings indicate that concurrent auditory input does not affect all readers similarly, but may depend on their baseline processing efficiency. Although no conclusions can be drawn about its influence on local ambiguity resolution, the results provide evidence that reading-while-listening may support older adults’ sentence reading, at least in Chinese.

## Figures and Tables

**Table 1:** Characteristics of the older adults in different conditions.

Condition	Age (year)	Formal Education (year)	Visual Acuity (log mar)	Digit Span Score	Vocabulary Score	MOCA Score
Reading-only	69.4 (3.5)	14.4 (1.9)	0.24 (0.06)	11.4 (1.6)	64.3 (5.6)	27.8 (1.3)
Reading-while-listening	68.9 (5.2)	14.1 (2.5)	0.26 (0.06)	11.9 (1.4)	66.5 (10.8)	27.4 (1.2)

**Table 2:** Example Sentence Stimuli.

Conditions	Target Word	Plausibility	Verb	Target Word	Example Sentence Materials
Control conditions	Two-character control word	Plausible	观察 survey	地形 terrain	侦察兵正准备观察***地形***获取敌军营地信息。 The scout is preparing to survey ***terrain*** to get information about the enemy camp.
	Two-character control word	Implausible	打开 open	地形 terrain	侦察兵正准备打开***地形***获取敌军营地信息。 The scout is preparing to open ***terrain*** to get information about the enemy camp.
Temporary semantic ambiguity Experimental conditions	Three-character incremental word	Plausible ^a^	观察 survey	地形图 topographic map	侦察兵正准备观察***地形图***获取敌军营地信息。 The scout is preparing to survey ***topographic map*** to get information about the enemy camp.
	Three-character incremental word	Implausible ^a^	打开 open	地形图 topographic map	侦察兵正准备打开***地形图***获取敌军营地信息。 The scout is preparing to open ***topographic map*** to get information about the enemy camp.

Note. Underlined words are the pre-target verbs. Bold italic words are the target words. ^a^ Plausibility refers specifically to the local semantic congruency between the verb and the initial two-character embedded word in the three-character incremental word, which constitutes a temporary ambiguity. Note that the control words were identical to the corresponding embedded word. Comprehension question example: “侦察兵是否准备获取敌军营地信息？”, meaning “Did the scout prepare to get information about the enemy camp?” The question’s answer is Yes.

**Table 3:** Characteristics of the Pre-target Verbs.

Characteristic	Plausible ^a^	Implausible ^a^	*t*	*p*
Word frequency	39 (43)	31 (45)	1.1	0.27
First-character frequency	518 (582)	522 (701)	0.23	0.81
Second-character frequency	619 (965)	846 (1304)	1.24	0.21
First-character stroke	7.9 (2.7)	8.2 (2.5)	0.78	0.43
Second-character stroke	7.6 (2.7)	7.8 (2.5)	0.17	0.85

Note. Word and character frequencies are reported in occurrences per million. The standard deviation of the mean is shown in parentheses. ^a^ Note that the labels indicate the local contextual plausibility of the verb and control two-character words. The three-character incremental words were always plausible.

**Table 4:** Mean eye movement measures for sentence level.

Measure	Older Adult		Young Adult	
	Reading-Only	Reading-While-Listening	Reading-Only	Reading-While-Listening
Sentence reading time (ms)	7784 (63)	5417 (37)	3583 (28)	3852 (21)
Average fixation duration (ms)	257 (1)	261 (1)	219 (1)	240 (1)
Number of fixations	26.70 (0.22)	17.71 (0.13)	13.71 (0.09)	13.63 (0.07)
Number of regressions	8.19 (0.10)	4.83 (0.07)	3.61 (0.04)	3.82 (0.03)
Average Forward Saccade Amplitude (char)	1.77 (0.01)	2.12 (0.01)	2.99 (0.01)	2.42 (0.01)

Note. Standard errors are given in parentheses.

**Table 5:** Summary of sentence-level statistical effects.

Age Group	Contrast	Statistic	Sentence Reading Time	Average Fixation Duration	Number of Fixations	Number of Regressions	Average Forward Saccade Amplitude
Age Group × reading condition		*b*	−0.43	−0.08	−8.92	−3.70	0.91
		*SE*	0.09	0.04	1.36	0.64	0.18
		*t*/*z*	−4.91 *	−2.07 *	−6.55 *	−5.80 *	4.96 *
		*p*	<0.001	0.04	<0.001	<0.001	<0.001
		95% CI	[−0.61, −0.26]	[−0.15, −0.00]	[−11.58, −6.25]	[−4.95, −2.45]	[0.55, 1.27]
		Cohen’s d	−1.84	−0.73	−1.65	−1.32	1.72
Older adults	(reading only vs. reading-while-listening)	*b*	0.34	−0.02	8.99	3.40	−0.35
		*SE*	0.07	0.03	1.04	0.49	0.14
		*t*/*z*	5.02 *	−0.54	8.62 *	6.96 *	−2.48 *
		*p*	<0.001	0.59	<0.01	<0.001	0.01
		95% CI	[0.21, 0.47]	[−0.07, 0.04]	[6.95, 11.04]	[2.45, 4.36]	[−0.63, −0.07]
		Cohen’s d	1.44	−0.15	1.66	1.21	−0.66
Young adults	(reading only vs. reading-while-listening)	*b*	−0.09	−0.09	0.08	−0.30	0.56
		*SE*	0.06	0.02	0.87	0.41	0.12
		*t*/*z*	−1.66	−3.86 *	0.09	−0.73	4.77 *
		*p*	0.10	<0.001	0.93	0.47	<0.001
		95% CI	[−0.21, 0.02]	[−0.14, −0.05]	[−1.63, 1.79]	[−1.10, 0.51]	[0.33, 0.80]
		Cohen’s d	−0.40	−0.88	0.02	−0.11	1.06
Reading-while-listening	(Older adults vs. Young adults)	*b*	−0.37	−0.09	−4.08	−1.06	0.31
		*SE*	0.06	0.03	0.93	0.44	0.13
		*t*/*z*	−6.11 *	−3.50 *	−4.39 *	−2.45 *	2.46 *
		*p*	<0.001	<0.001	<0.001	0.015	0.015
		95% CI	[−0.49, −0.25]	[−0.14, −0.04]	[−5.90, −2.26]	[−1.92, −0.21]	[0.06, 0.55]
		Cohen’s d	−1.57	−0.85	−0.76	−0.38	0.58
Reading-only	(Older adults vs. Young adults)	*b*	−0.80	−0.16	−12.99	−4.77	1.22
		*SE*	0.06	0.03	1.00	0.47	0.13
		*t*/*z*	−12.42 *	−6.09 *	−13.06 *	−10.21 *	9.08 *
		*p*	<0.001	<0.001	<0.001	<0.001	<0.001
		95% CI	[−0.93, −0.67]	[−0.22, 0.11]	[−14.94, −11.04]	[−5.68, −3.85]	[0.96, 1.48]
		Cohen’s d	−3.41	−1.58	−2.41	−1.70	2.30

Note. Asterisks indicate statistically significant effects, *p* < 0.05. Converged model for SRT, AFD, NR, AFSA, NF: depvar.lmer = lmer(depvar ~ MODALITY * AGE + (1|participant) + (1|item), datafile).

**Table 6:** Mean Eye Movement Measures for Target Regions.

Measure	Control Target Word				Ambiguous Target Word			
	Older Adult		Young Adult		Older Adult		Young Adult	
	Plausible	Implausible	Plausible	Implausible	Plausible ^a^	Implausible ^a^	Plausible ^a^	Implausible ^a^
Reading-only								
First-fixation duration	271 (4)	279 (4)	235 (3)	239 (3)	268 (4)	268 (4)	227 (3)	223 (3)
Gaze duration	372 (8)	402 (10)	273 (5)	281 (5)	516 (12)	522 (13)	322 (6)	327 (6)
Total reading time	692 (18)	872 (25)	407 (10)	459 (11)	960 (22)	959 (22)	489 (12)	478 (11)
Regressions-in (%)	35 (2)	43 (2)	24 (2)	28 (2)	41 (2)	38 (2)	26 (1)	24 (1)
Reading-while-listening								
First-fixation duration	283 (4)	293 (4)	251 (3)	262 (3)	279 (4)	283 (4)	254 (3)	263 (3)
Gaze duration	350 (6)	360 (6)	294 (4)	311 (5)	449 (7)	470 (8)	371 (6)	374 (6)
Total reading time	528 (12)	601 (13)	442 (7)	515 (10)	671 (14)	680 (12)	577 (10)	573 (9)
Regressions-in (%)	26 (2)	32 (2)	33 (2)	37 (2)	29 (2)	26 (2)	40 (1)	39 (1)

Note. Fixation time measures are shown in ms. Standard errors are given in parentheses. ^a^ Note that the labels indicate the plausibility of the embedded words. The incremental words were always plausible.

**Table 7:** Summary of Statistical Effects for Target Regions.

			Reading-Only				Reading-While-Listening			
Age Group	Contrast	Statistic	FFD	GD	TRT	RI	FFD	GD	TRT	RI
Older	Control word plausibility	*b*	−0.02	−0.04	−0.21	−0.38	−0.03	−0.01	−0.11	−0.30
		*SE*	0.02	0.02	0.03	0.12	0.02	0.02	0.03	0.12
		*t*/*z*	−0.81	−1.85	−7.34 *	−3.21 *	−1.69	−0.56	−4.14 *	−2.41 *
		*p*	0.421	0.065	<0.001	0.001	0.092	0.576	<0.001	0.016
		95% CI	[−0.05, 0.02]	[−0.09, 0.00]	[−0.26, −0.15]	[−0.95, −0.70]	[−0.07, 0.00]	[−0.06, 0.03]	[−0.17, −0.06]	[−0.61, −0.15]
		Cohen’s d	−0.05	−0.10	−0.41	−0.38	−0.09	−0.03	−0.22	−0.30
Young	Control word plausibility	*b*	−0.01	−0.02	−0.10	−0.24	−0.03	−0.05	−0.13	−0.20
		*SE*	0.02	0.02	0.03	0.12	0.02	0.02	0.02	0.10
		*t*/*z*	−0.54	−1.06	−3.73 *	−2.00	−2.16	−2.65 *	−5.59 *	−2.01
		*p*	0.587	0.290	<0.001	0.045	0.031	0.008	<0.001	0.044
		95% CI	[−0.04, 0.02]	[−0.07, 0.02]	[−0.15, −0.05]	[−0.54, −0.06]	[−0.06, −0.00]	[−0.09, −0.01]	[−0.18, −0.09]	[−0.48, −0.01]
		Cohen’s d	−0.03	−0.05	−0.19	−0.24	−0.10	−0.12	−0.26	−0.20
Older	Embedded word plausibility in incremental words	*b*	−0.00	−0.00	0.00	0.15	−0.02	−0.05	−0.04	0.12
		*SE*	0.02	0.03	0.03	0.12	0.02	0.02	0.03	0.12
		*t*/*z*	−0.21	−0.16	0.04	1.32	−1.00	−2.01	−1.42	0.97
		*p*	0.837	0.875	0.971	0.188	0.316	0.045	0.155	0.334
		95% CI	[−0.04, 0.03]	[−0.05, 0.05]	[−0.05, 0.05]	[−0.07, 0.38]	[−0.05, 0.02]	[−0.10, −0.00]	[−0.09, 0.01]	[−0.12, 0.35]
		Cohen’s d	−0.01	−0.01	0.00	0.15	−0.05	−0.10	−0.07	0.12
Young	Embedded word plausibility in incremental words	*b*	0.02	−0.01	0.02	0.09	−0.03	−0.01	−0.01	0.01
		*SE*	0.02	0.02	0.02	0.12	0.01	0.02	0.02	0.09
		*t*/*z*	1.46	−0.48	0.65	0.81	−2.11	−0.25	−0.46	0.09
		*p*	0.144	0.634	0.515	0.419	0.035	0.800	0.649	0.926
		95% CI	[−0.01, 0.05]	[−0.05, 0.03]	[−0.03, 0.06]	[−0.13, 0.32]	[−0.06, 0.00]	[−0.04, 0.03]	[−0.05, 0.03]	[−0.18, 0.19]
		Cohen’s d	0.07	−0.02	0.03	0.09	−0.09	−0.01	−0.02	0.01

Note. FFD = first-fixation duration; GD = gaze duration; TRT = total reading time; RI = probability of a regression-in; SE = standard error. Asterisks indicate effects significant at the Bonferroni-corrected threshold (*p* < 0.017). Effects significant at the uncorrected threshold (*p* < 0.05) but not surviving Bonferroni correction are treated as exploratory observations and are not marked. Converged model for FFD, GD, TRT: depvar.lmer = lmer(depvar ~ MODALITY * PI * AGE + (1|participant) + (1|item), datafile). Converged model for RI: depvar.glmer13 = glmer(depvar ~ MODALITY * PI * AGE + (1|participant) + (1|item), datafile, family = binomial).

## Data Availability

The data in this study are available from https://osf.io/94zpu (accessed on 1 May 2026).

## Supplementary Materials

The following supporting information can be downloaded at: https://www.mdpi.com/article/10.3390/bs16061020/s1, Figure S1: Power Curve for the Age × Reading Condition Interaction; Figure S2: Sensitivity Analysis for the Age × Reading Condition Interaction; Table S1: a direct comparison of Cohen’s d values for the older vs. young adults contrast across reading conditions; Section S1: Audio Duration and Response Timing.

## Abbreviations

The following abbreviations are used in this manuscript:

FFD	First fixation duration
GD	Gaze duration
TRT	Total reading time
RI	Regression-in
SRT	Sentence reading time
AFD	Average fixation duration
NF	Number of fixations
NR	Number of regressions
AFS	Average length of forward-directed saccades
