# Supplementary material for: Does Reading-While-Listening Facilitate Reading in Older Adults? Evidence from Eye Movements
Source: Behav Sci (Basel). 2026 Jun 18;16(6):1020. doi: 10.3390/bs16061020 (PMC13296048; doi:10.3390/bs16061020)
Supplement: Supplementary file 1 [file behavsci-16-01020-s001.zip › behavsci-4327708-supplementary.pdf]

Supplementary Materials

Supplementary Figure S1: Power Curve for the Age × Reading Condition Interaction

Power curve for the Age × Reading Condition interaction effect, was estimated via parametric simulation using the *simr* package (Green & MacLeod, 2016). Models were constructed using the makeLmer function based on fixed effects, random effect variances, and residual variance extracted from the fitted linear mixed-effects model. Sample sizes were expanded from a total of N = 24 to N = 188, balanced across four cells (Older Adults - reading-while-listening, Older Adults - reading-only, Young Adults - reading-while-listening, Young Adults - reading-only). Each data point represents power estimated from 1,000 simulations. Error bars indicate 95% confidence intervals. The dashed horizontal line indicates the 80% power threshold. Results indicate that a minimum total sample of 24 participants (6 per cell) is sufficient to achieve 80% power (82.1%, 95% CI [79.6%, 84.4%]) for detecting the interaction effect. The actual sample used in the present study (N = 183) substantially exceeds this minimum requirement.

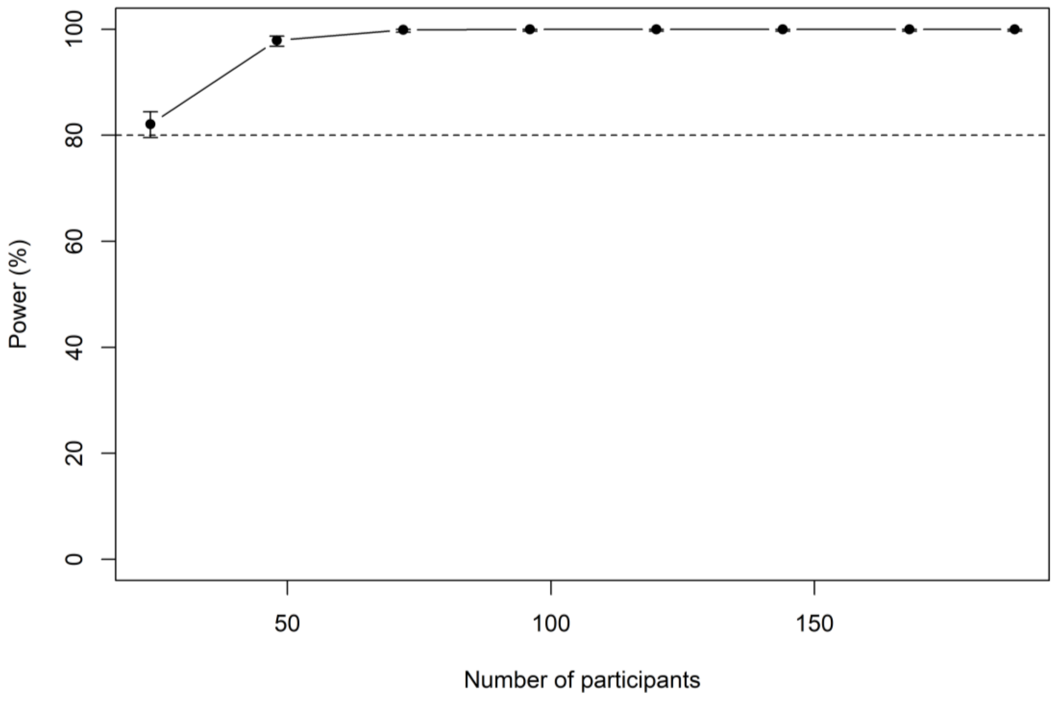

Figure S1. Power Curve for the Age × Reading Condition Interaction

Supplementary Figure S2: Sensitivity Analysis for the Age × Reading Condition Interaction

Sensitivity curve for the Age × Reading Condition interaction effect. The curve shows estimated statistical power across a range of effect sizes (Cohen's *d*) given the actual sample sizes in the present study (Older Adults: n = 39 for the reading-while-listening condition, n = 36 for the reading-only condition; Young Adults: n = 60 and n = 48, respectively; items: n = 80). Each data point represents power estimated from 500 simulations. Error bars indicate 95% confidence intervals. The dashed red and blue horizontal lines indicate the 80% and 95% power thresholds, respectively. The vertical green dashed line marks the observed interaction effect size (d = 1.84). Results indicate that the current sample can reliably detect effects as small as d = 1.10 at 80% power and d = 1.40 at 95% power.

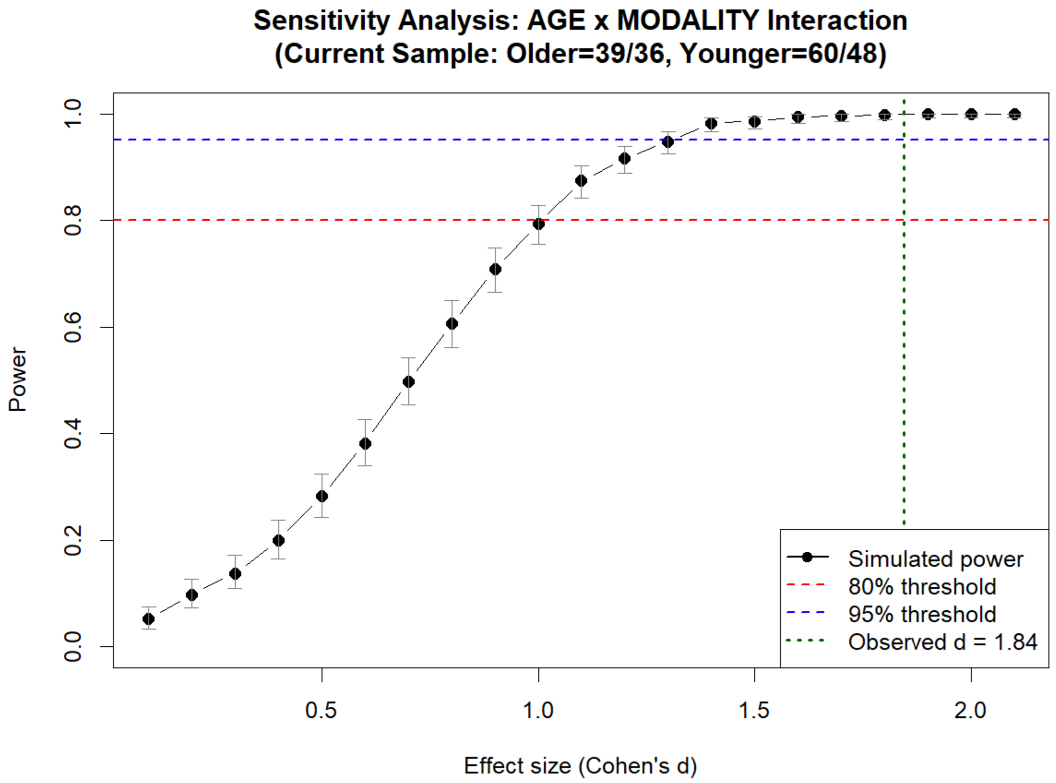

Figure S2. Sensitivity Analysis for the Age × Reading Condition Interaction

Supplementary Table S1: a direct comparison of Cohen's d values for the Older vs. Young Adults contrast across reading conditions

Measure	Reading-only |d|	RWL |d|	Reduction (%)	Direction of age gap
Sentence reading time (SRT)	3.41	1.57	**54%**	Older > Younger
Average fixation duration (AFD)	1.58	0.85	**46%**	Older > Younger
Number of fixations (NF)	2.41	0.76	**68%**	Older > Younger
Number of regressions (NR)	1.70	0.38	**78%**	Older > Younger
Average forward saccade amplitude (AFSA)	2.30	0.58	**75%**	Younger > Older

Note. Cohen’s d values represent the standardized mean difference between Older Adults and Young Adults within each reading condition, derived from the linear mixed-effects models reported in Table 5 of the main text. Absolute values of d are presented to facilitate comparison of gap magnitude. Reduction (%) = (Reading-only |d| − RWL |d|) / Reading-only |d| × 100. RWL = reading-while-listening. AFSA reflects saccade length (larger values indicate longer saccades, a marker of more efficient reading); the direction of the age gap is reversed for this measure.

To quantify the extent to which the reading-while-listening condition reduced age-related performance differences, we compared Cohen's d values for the Older Adults versus Young Adults contrast separately for the reading-only and RWL conditions across all five sentence-level eye-movement measures (see Supplementary Table S1).

Cohen's d values were derived from the linear mixed-effects models reported in Table 5 of the main text.

As shown in Supplementary Table S1, the age-related performance gap was substantially reduced under the RWL condition relative to the reading-only condition across all measures. The largest reductions were observed for Number of Regressions (78% reduction; reading-only: |d| = 1.70, RWL: |d| = 0.38) and Average Forward Saccade Amplitude (75% reduction; reading-only: |d| = 2.30, RWL: |d| = 0.58),

followed by Number of Fixations (68% reduction; reading-only: $|d| = 2.41$, RWL: $|d| = 0.76$). Sentence Reading Time showed a 54% reduction (reading-only: $|d| = 3.41$, RWL: $|d| = 1.57$), and Average Fixation Duration showed a 46% reduction (reading-only: $|d| = 1.58$, RWL: $|d| = 0.85$). Notably, even after the RWL condition substantially reduced these gaps, age-related differences remained statistically significant and large in magnitude across all measures, indicating that the concurrent auditory input partially but not fully compensated for age-related declines in reading efficiency.

Taken together, these results provide quantitative evidence that age-related differences were substantially smaller in the RWL condition than in the reading-only condition, reducing the age-related performance gap by 46–78% across all sentence-level eye-movement measures. This pattern is consistent with the interpretation that concurrent auditory input provides compensatory support for older adults' reading, while having comparatively little impact on younger adults' already-efficient reading behavior.

Supplementary Section S1: Audio Duration and Response Timing

The mean duration of the 80 experimental sentences was 4630 ms (*SD* = 567 ms). To examine the temporal relationship between reading completion and audio playback, we calculated the proportion of trials on which the response key was pressed before versus after audio offset. Older adults pressed the response key before audio offset on 37% of trials and after audio offset on 63% of trials. Young adults pressed the response key before audio offset on 72% of trials and after audio offset on 28% of trials. These results indicate that older adults more frequently continued reading after the audio had finished, whereas young adults more frequently completed reading while the audio was still playing.
